# Concentrations and Risk Assessments of Antibiotics in an Urban–Rural Complex Watershed with Intensive Livestock Farming

**DOI:** 10.3390/ijerph182010797

**Published:** 2021-10-14

**Authors:** Hyun-Jeoung Lee, Deok-Woo Kim, Chansik Kim, Hong-Duck Ryu, Eu-Gene Chung, Kyunghyun Kim

**Affiliations:** 1 Department of Water Environment Research, National Institute of Environmental Research (NIER), Hwangyoung-ro 42, Seo-gu, Incheon 22689, Korea; leehj2018@korea.kr (H.-J.L.); dwkim83@korea.kr (D.-W.K.); nanumenv@korea.kr (H.-D.R.); matthias@korea.kr (K.K.); 2 Accident Coordination & Training Division, National Institute of Chemical Safety, Gajeongbuk-ro 90, Yuseong-gu, Dajeon 34111, Korea; chansik83@korea.kr

**Keywords:** veterinary antibiotics, livestock excreta, high-resolution mass spectrometry, urban-rural complex pollution sources, risk quotient

## Abstract

Antibiotics used for the treatment of humans and livestock are released into the environment, whereby they pose a grave threat to biota (including humans) as they can cause the emergence of various strains of resistant bacteria. An improved understanding of antibiotics in the environment is thus vital for appropriate management and mitigation. Herein, surface water and groundwater samples containing antibiotics were analyzed in an urban–rural complex watershed (Cheongmi Stream) comprising intensive livestock farms by collecting samples across different time points and locations. The spatiotemporal trends of the residual antibiotics were analyzed, and ecological and antibiotic resistance-based risk assessments were performed considering their concentrations. The results showed that the concentrations and detection frequencies of the residual antibiotics in the surface water were affected by various factors such as agricultural activities and point sources, and were higher than those found in groundwater; however, frequent detection of antibiotics in groundwater showed that residual antibiotics were influenced by factors such as usage pattern and sewage runoff. Furthermore, few antibiotics posed ecological risks. The risk assessment methods adopted in this study can be applied elsewhere, and the results can be considered in the environmental management of residual antibiotics in the Cheongmi Stream watershed.

## 1. Introduction

Antibiotics are deemed indispensable for the protection of human life, and both their production and usage continue to increase steadily [1,2,3]. Furthermore, the consumption of meat has increased with the improved standards of living, and thus livestock breeding facilities have been scaled up in numerous regions to meet the growing demand [2,4,5]; consequently, considerable quantities of antibiotics are being used to prevent and treat diseases of livestock and to promote growth. According to the Korea Health Industry Development Institute, the quantity of national antibiotic production has continuously increased, and the market size was estimated at approximately KRW 1.1 trillion (USD ~950 million) in 2015 [1,6]. Accordingly, the use of antibiotics for humans and animals has also increased, with ~1000 tons of antibiotics sold strictly for animals in Korea in 2018 [7].

Substantial amounts of antibiotics consumed by humans or those administered to livestock during breeding are discharged into the environment via excreta through various routes such as compost, liquid manure, and runoff from breeding facilities. Subsequently, such antibiotics flowing into the environment have emerged as a new global concern due to their potential adverse effects [4,8]. Many studies have suggested that antibiotics present at concentrations of several ng·L^−1^ to μg·L^−1^ can inhibit the growth of aquatic organisms, plants, and humans [9,10,11]. Antibiotics in the environment can be toxic, and can promote the occurrence of resistant bacteria, leading to the occurrence of potentially disastrous problems. Based on previous studies, deaths attributed to infections with antibiotic-resistant bacteria have been estimated to be ~700,000 a year [12,13,14].

Globally, the standards for the analysis and management of residual antibiotics in environmental media are insufficient; thus, it is necessary to evaluate their status [2,3,4]. The concentrations of antibiotics in the environment are known to be affected by various factors such as the pollution source, geography, climate, and usage patterns [3,15,16]; therefore, it is crucial to monitor and analyze residual antibiotics in environmental media across space and time to understand their statuses in different watersheds and to establish efficient management methods to mitigate their environmental effects.

Antibiotics are developed to induce biological effects, and can exert a wide range of impacts on ecosystems once released into the environment; hence, it is essential to evaluate their risks [3,17]. Many studies have conducted ecological risk assessment of antibiotics, and it has been reported that a few antibiotic classes pose potential risks [18,19,20]. In general, risk quotient (RQ) values are widely used for ecological risk assessment, which is calculated by dividing the maximum residual concentration by the predicted no effective concentration (PNEC) [3,19,21]. Zhang et al. (2020) reported that most antibiotics had no risk; a study has reported the possibility that bacteria may develop antibiotic resistance even under low concentrations of antibiotics [8,19]. Therefore, further ecological risk assessments must include the risk posed by the resistant bacteria, as they can lead to fatalities in both humans and other biota [8,19,22].

Therefore, the Cheongmi Stream watershed was selected for the present study, which encompassed an urban–rural area with dense livestock breeding facilities. Spatiotemporal trends and current statuses were examined for nine antibiotics in stream surface water and groundwater samples. Furthermore, ecological and antibiotic resistance-based risk assessments were performed by determining the residual concentrations of the target antibiotics. This study aimed to provide a more complete understanding of the risk assessment methods adopted for ascertaining the extent of the contamination of natural waters by antibiotic-resistant bacteria to help ensure the prolonged health of ecosystems and human populations.

## 2. Materials and Methods

### 2.1. Study Area

Anseong City is an urban–rural complex in the southernmost part of Gyeonggi-do, South Korea, with ~180,000 residents. The Cheongmi Stream watershed is one of the eleven watersheds in Anseong City, and the corresponding number of livestock and quantity of livestock excreta produced in the watershed are 770,000 heads and 900 tons·d^−1^, as reported in the year 2018, respectively [23,24]. The Cheongmi Stream watershed consists of 54% agricultural land, 30% forest, and 9% urban area. Four on-site swine wastewater treatment facilities (OSWTF; treatment capacity, 18.9–72.2 m^3^·d^−1^), three village sewage treatment facilities (VSTF; 33–150 m^3^·d^−1^), and two municipal sewage treatment plants (MSTP; 2200 and 3000 m^3^·d^−1^) are present within the watershed [25,26,27,28]. This study area was selected for its importance in terms of water pollution management, as it is located upstream of the Han River, an important water source for Seoul, the largest metropolis in South Korea.

**Figure 1:**
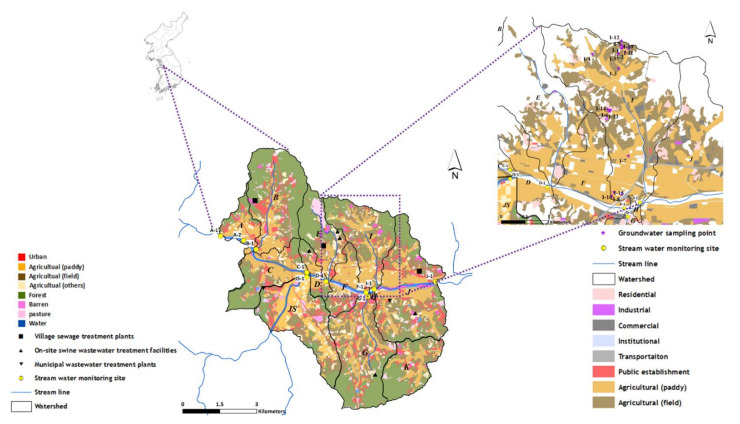
Map showing the sampling points. The alphabet symbols in the watershed map indicate the sampled subwatersheds.

The Cheongmi Stream watershed consists of 18 subwatersheds, and a total of 10 sampling points were considered in nine of these subwatersheds. Of the ten sampling points, six were located at the end of the mainstream (A-1, A-2, C-1, D-1, F-1, and J-1), and four at the end of tributaries (B-1, JS-1, G-1, and I-1). Surface water samples were collected in March, June, August, and December 2018, whereas groundwater samples were collected in March (13 points), June (12 points), and August (10 points) excluding the winter (December) when the groundwater wells are not used in the watershed I; this maintained the highest risk of pollution among the subwatersheds analyzed. Sampling points and land-cover information of the Cheongmi Stream watershed are shown in Figure 1.

### 2.2. Chemicals and Materials

To quantify antibiotics in the environmental water samples, nine standard and seven internal standard (ISTD) chemicals were used. The ISTD chemicals with similar structures or retention times in the ultra-high-performance liquid chromatography-high-resolution mass spectrometry (UHPLC-HRMS) were selected. Further details of the chemicals, materials (Text A1), and sample preparation methods (Text A2) can be found in the Appendix A.

### 2.3. Analysis

The residual antibiotics in the environmental water were extracted and analyzed using the Thermo Scientific Dionex Ultimate 3000 system (Thermo Scientific Fisher, Bremen, Germany) combined with an online solid phase extraction (SPE) system (EQuan Max Plus, Thermo Scientific, San Jose, CA, USA). For this analysis, the methods reported by Kim et al. (2018) were performed [2]. A Hypersil GOLD aQ column and Reversed Xbridge C18 column (2.1 mm × 50 mm i.d., 2.5 μm, Waters, Milford, MA, USA) were used to extract and analyze antibiotics in the samples, respectively. The total analysis time was 30 min and 5 mL of the sample was injected into the online SPE system for 5 min. The detailed equipment conditions for antibiotic extraction and analysis are listed in Table A1. The Xcalibur 4.0 (Thermo Fisher Scientific) software was used for the control and data collection. Information on the analysis and quality assurance (Text A3) and quality control methods (Text A4 and Table A2) used can be found in the Appendix A.

### 2.4. Risk Assessment

To calculate the risk quotient (RQ), the maximum concentrations of antibiotics for each sampling time and point were divided by the predicted no-effect concentration (PNEC) [3,19,29], and the PNEC values derived by Lee (2021) were used for ecological risk assessment [5]. Additionally, the PNEC values for the antibiotic resistance risk assessment reported in many studies were used [19,22,30]. The two types of PNEC values obtained for the antibiotics are shown in Table 1.

## 3. Results and Discussion

### 3.1. Occurrence of Antibiotics

**Figure 2:**
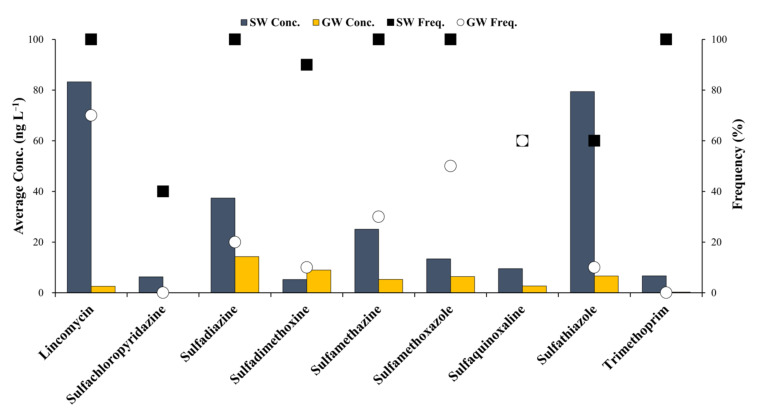
Concentrations and detection frequencies of residual antibiotics in the Cheongmi Stream watershed. Dark blue bar: concentrations of antibiotics in the surface water, yellow bar: concentration of antibiotics in groundwater, square: detection frequency of antibiotics in the surface water, circle: detection frequency of antibiotics in the groundwater.

The residual concentrations of antibiotics in the surface water and groundwater samples of the Cheongmi Stream watershed are shown in Figure 2, and the 1-year average concentrations obtained at each sampling point are presented to explain the trends and concentrations of the target antibiotics analyzed. Specific residual concentrations for the antibiotics obtained at each sampling point are shown in Table A3 and Table A4.

The concentrations and detection frequencies of the antibiotics that were identified in surface water samples were higher than those obtained for the groundwater samples. The average concentration of LIN—estimated to be 83.3 ng·L^−1^—was the highest concentration evaluated, and was detected at all sampling sites. Additionally, SDZ, SMZ, SMX, and TMP were also detected at each sampling point. The maximum residual concentrations of STZ and SDZ were relatively high (79.4 and 37.4 ng·L^−1^, respectively), whereas SCP, SDX, SQX, and STZ were detected in points 4, 9, and 6, respectively, suggesting that trends in the use of antibiotics varied by location.

Chen et al. (2020) reported that the residual concentration of LIN in the Xiangjiang River was MQL–12.2 ng·L^−1^ [31]. The values obtained in the present study were higher, most likely because of the lower distances between the sampling points and pollution sources discharging antibiotics. Furthermore, the detection frequency and maximum residual concentration of SDZ in the river waters of Beijing, Changzhou, and Shenzhen measured by Wang et al. (2015) were 91.7% and 344 ng·L^−1^, respectively, which were greater than those observed in this study [32].

Conversely, SCP was not detected in the groundwater sample obtained at any sampling point, and the average residual concentration of antibiotics in the groundwater was ≤10 ng·L^−1^ (except for SDZ). As observed with the surface water samples, LIN showed the highest groundwater detection frequency (70%), but its average concentration was a fraction of that noted above (2.6 ng·L^−1^). Hu et al. (2010) analyzed the residual concentrations of antibiotics for animals in livestock excreta, soils, vegetables, and groundwater near an organic vegetable production area of Northern China, and reported that the residual concentration of LIN in the groundwater was below the detection limit (BDL; 8.3 ng·L^−1^), similar (albeit slightly higher) to the values observed in the present study [33]. Likewise, the concentrations of SDZ obtained and reported by Yao et al. (2011) ranged from BDL–16.7 ng·L^−1^, which was similar to the value reported herein [34].

### 3.2. Temporal Variation

#### 3.2.1. Surface Water

**Figure 3:**
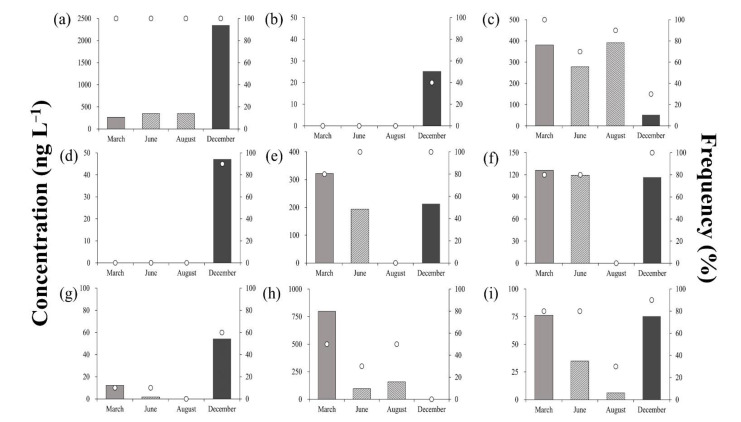
Residual concentrations and detection frequencies of antibiotics in surface water during the sampling campaigns; (**a**) Lincomycin, (**b**) Sulfachloropyridazine, (**c**) Sulfadimethoxine, (**d**) Sulfadiazine, (**e**) Sulfamethazine, (**f**) Sulfamethoxazole, (**g**) Sulfaquinoxaline, (**h**) Sulfathiazole, (**i**) Trimethoprim.

Figure 3 shows the average residual concentrations and detection frequencies of antibiotics in the surface water sample throughout the sampling campaign. As mentioned, LIN was the only target antibiotic with 100% detection frequency, maintaining an average seasonal detection concentration range of 26.5–234.7 ng·L^−1^. Concentrations obtained in December were 6.7–8.9 times higher than those obtained in March–August (Figure 3a), indicating that the seasonal flow of LIN into the environment during this period was the highest. LIN is an antibiotic used for the treatment of diseases caused by *Staphylococcus* and *Streptococcus* in people, and to prevent or treat various diseases such as mycoplasmas and microbials in livestock such as chicken and pigs [35,36,37]. During the seasonal change from September to November in South Korea, LIN is used as an injection or feed additive to prevent sickness in livestock due to the stressors associated with the changing weather [38], most likely driving the elevated concentrations observed in the surface water.

SCP and SDX were not detected from March to August; however, they were detected at low concentrations in December (6.3 and 5.3 ng·L^−1^, respectively; Figure 3b,c). As microbial activity water systems were positively correlated with temperature, the degree of degradation of antibiotics also decreased in the lower temperatures recorded in December [39]. Detection frequencies also differed for SCP and SDX (40% and 90%, respectively), but there was a possibility that both were used similarly due to their lone detection in winter.

The average concentration of SDZ was <50 ng·L^−1^ across all seasons, peaking in August (43.5 ng·L^−1^), and reaching its minimum in December (17.3 ng·L^−1^; Figure 3d). Detection frequency was 100% in March, but was only 30% in December (three out of 10 points). Wei et al. (2011) reported a SDZ detection frequency and concentration of 55% and 1000 ng L^−1^, respectively [40]. Furthermore, their observed environmental SDZ concentrations varied across a wide range of BDL–1.06 ng·L^−1^, although it was generally higher than the concentrations obtained in the present study [18,31,41].

The concentration ranges of SMZ, SMX, SQX, STZ, and TMP were BDL-40.2, BDL-15.8, BDL-12.1, BDL-159.5, and 2.0−9.5 ng·L^−1^, respectively. No antibiotic, except for STZ, exceeded 50 ng·L^−1^, and TMP demonstrated low concentrations < 10 ng·L^−1^ across all sampling campaigns. STZ showed a relatively high concentration in March (159.5 ng·L^−1^), but concentrations decreased significantly in June (31.9 ng·L^−1^). The five antibiotics showed a similar trend, indicating that precipitation was most likely the predominant mechanism driving the observed patterns. Precipitation levels in June and August of 2018 were higher than those of March recorded in the same year (the maximum monthly rainfall in 2018 was observed in August). Consequently, the concentrations of antibiotics tended to decrease due to the dilution effect, with the limited detection of antibiotics in August. Moreover, sulfonamide-based and sulfonamide-containing materials are known to undergo photolysis. As the intensity of sunshine in August was greater than that observed in other periods, photolysis most likely further contributed to the decreased concentrations of antibiotics during this period. Consequently, it was confirmed that the climate conditions such as precipitation remarkably affected the residual concentration of antibiotics in surface water.

#### 3.2.2. Groundwater

**Figure 4:**
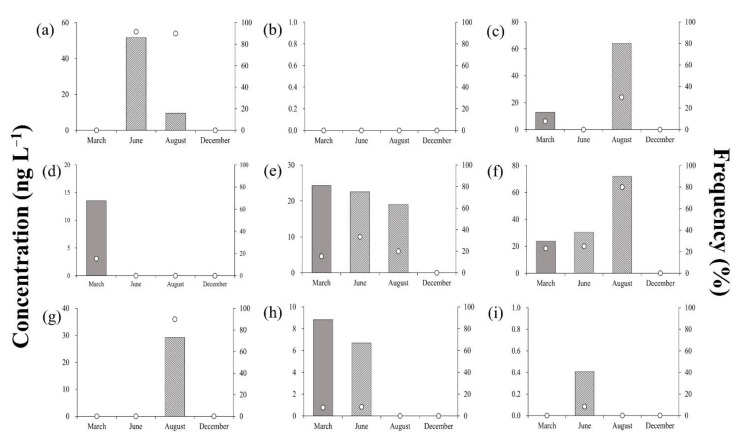
Residual concentrations and detection frequencies of antibiotics in surface water during the sampling campaigns: (**a**) Lincomycin, (**b**) Sulfachloropyridazine, (**c**) Sulfadimethoxine, (**d**) Sulfadiazine, (**e**) Sulfamethazine, (**f**) Sulfamethoxazole, (**g**) Sulfaquinoxaline, (**h**) Sulfathiazole, (**i**) Trimethoprim.

Figure 4 shows the residual concentrations and detection frequencies of antibiotics in groundwater samples throughout the sampling campaigns, averaging < 30 ng·L^−1^, and values were significantly lower than those recorded in the surface water samples. Particularly, the residual concentrations of SCP, SDX, SQX, STZ, and TMP were <10 ng·L^−1^. SDZ was detected in relatively high concentrations (except for in June), averaging 12 ng·L^−1^ in March and 21.3 ng·L^−1^ in August.

Balzer et al. (2016) reported that antibiotics entered surface waters through the effluents discharged from sewage treatment plants, sewage sludge, soil, groundwater, livestock excreta, and fertilizers containing livestock excreta; subsequently, pollutants from the surface water entered groundwater through the soil [42]. Accordingly, the observed concentrations and frequencies of antibiotics in the groundwater were lower than those in the surface water since the former could initially enter through other filtration and dilution media such as soils and streams.

### 3.3. Spatial Variation of Antibiotics in the Cheongmi Stream Watershed

#### 3.3.1. Spatial Variations in Surface Water

**Figure 5:**
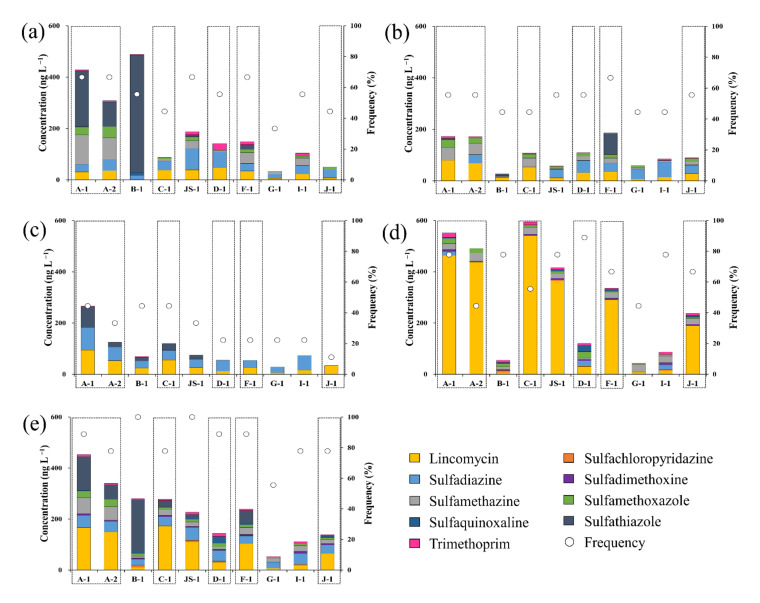
Residual cumulative concentrations of antibiotics at each sampling point during various months: (**a**) March, (**b**) June, (**c**) August, (**d**) December, (**e**) Average concentrations of samples from March to December; Yellow: lincomycin, Orange: SCP, Sky blue: SUL, Violet: SDX, Grey: SMZ, Green: SMX, Dark blue: SQX, Black: STZ, Pink: TMP, circle: frequency, and box: main stream.

Figure 5 shows the estimated concentrations of antibiotics in the surface water samples of each sampling point along the Cheongmi Stream by sampling period. The cumulative concentrations of antibiotics at each sampling point in March ranged from 33.6 to 488.2 ng·L^−1^, peaking at B-1, and reaching its minimum at G-1. At A-1, A-2, JS-1, and F-1, six antibiotics were detected, exhibiting a higher variety than the other sampling points. At B-1, the concentration of STZ was 457.0 ng·L^−1^, or was 93.5% of the total concentration, whereas STZ was also detected in high concentrations at A-1, A-2, and B-1, corresponding to the upstream sampling points. In contrast, SDZ was mainly detected at five points (JS-1, D-1, G-1, I-1, and J-1), notably excluding the three upstream sites, C-1, and F-1, thus corroborating that the use of antibiotic types varied by subwatershed. Furthermore, elevated concentrations of STZ were primarily detected upstream and were found to decrease downstream due to the lack of new inflow. The June sampling points with the highest and lowest cumulative concentrations of antibiotics were F-1 (186.0 ng·L^−1^) and B-1 (27.7 ng·L^−1^), respectively, with the concentrations of antibiotics decreasing between 1.2–17.7 times at the six sites (excluding C-1, G-1, F-1, and J-1).

The cumulative concentration of antibiotics at each point in August was <100 ng·L^−1^, except for that recorded in the A and C subwatersheds (Figure 5c), with the cumulative concentrations of A-1, B-1, C-1, and JS-1 increasing between 1.1–2.5 times over the levels recorded in June. The cumulative concentrations of the six other sampling points decreased between 1.2–3.4 times.

Among the four sampling campaigns, all antibiotics were detected in December (except for STZ; Figure 5d). Although STZ was detected consistently from March to August, its notable absence in December indicated that its quantity of use decreased during the winter. Increasing cumulative concentrations for each sampling point seems to be primarily driven by the increasing LIN concentrations; for example, the concentrations reported in December significantly increased between 1.4–10.8 compared to those reported in August. The seasonal concentrations demonstrated the greatest level of change at F-1, from 26.9 to 291.2 ng·L^−1^, implying that the discharge of LIN in December might have been the most intensive at this site.

The average residual concentrations for each antibiotic obtained through the four seasonal sampling campaigns are shown in Figure 5e. In 2018, the cumulative concentrations of antibiotics at the 10 sampling points within the watershed ranged from 53.1 to 452.9 ng·L^−1^. Although the concentrations of individual antibiotics varied, the overall cumulative concentrations tended to decrease with streamflow from A-1 to D-1. At the tributary points (B-1 and JS-1), the dilution effect decreased the concentrations due to the higher flow rates, even when the concentrations of antibiotics that flowed directly into the downstream points were similar to those that directly entered upstream. In contrast, the cumulative concentration at F-1 reported in 2018 was 240.0 ng·L^−1^, which was 1.7 times greater than that recorded at D-1 (144.2 ng·L^−1^); thus, it was inferred that abundant antibiotics were discharged from subwatershed F. By landcover, farmland in subwatershed F occupied 58.1% of the territory, the highest among the 10 subwatersheds. Within these areas and in other farmlands, fertilizer containing livestock excreta and liquid manure are sprayed. Accordingly, the concentration of antibiotics in subwatershed F increased as these antibiotics flowed into the stream via external driving factors such as the runoff and leaching of antibiotics present in the compost and liquid fertilizers. Furthermore, the upper region of F-1 has two OSWTF and one VSTF, both of which are major polluting sources that discharge antibiotics to the environment. A similar phenomenon could also be observed in watershed J, where the concentration of antibiotics at J-1 was greater than that at I-1, and this was likely attributable to the increases from the local VSTF and MSTP. Consequently, it seems that antibiotic contamination by surface watersheds is greatly affected by pollution sources.

#### 3.3.2. Spatial Variations in Groundwater

**Figure 6:**
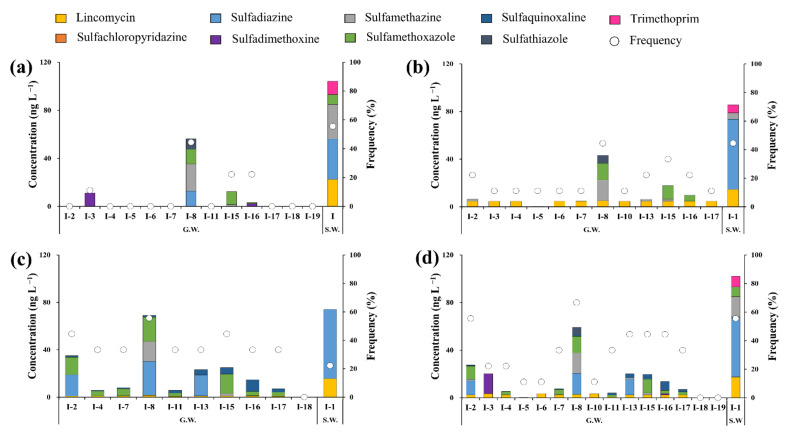
Residual cumulative concentrations of antibiotics in samples collected at each sampling point in (**a**) March, (**b**) June, (**c**) August, (**d**) Average concentrations of samples collected from March to August; Yellow: lincomycin, Orange: Sulfachloropyridazine, Sky blue: Sulfadiazine, Purple: Sulfadimethoxine, Grey: Sulfamethazine, Green: Sulfamethoxazole, Dark blue: Sulfaquinoxaline, Black: Sulfathiazole, Pink: Trimethoprim, Circle: frequency.

Figure 6 shows the cumulative antibiotic concentrations in groundwater samples for each sampling point in the Cheongmi Stream watershed. Figure 6a–d shows the values separately for each sampling period (March, June, August) as well as the cumulative average concentrations, respectively.

In March, antibiotics were detected at only four points (I-3, I-8, I-15, and I-16), and the cumulative concentrations ranged from 3.3 (I-16) to 56.3 (I-8) ng·L^−1^. Four antibiotics were detected at I-8, where both the cumulative concentration and number of antibiotics peaked. The cumulative concentrations detected in the surface water sample over the same period for watershed I were notably higher (104.1 ng·L^−1^). Furthermore, LIN, SDZ, SMZ, SMX, and TMP were detected in the surface water samples, whereas neither LIN nor TMP were detected in the groundwater.

The cumulative concentrations of antibiotics in the surface water and groundwater samples of watershed I were <100 ng·L^−1^ (Figure 6b). Unlike the measurements recoded in March, wherein antibiotics were only detected at four sampling points, ≤1 types of antibiotics were detected at I-11, I-18, and I-19 in June. The cumulative concentration during this period was the highest for I-8 (43.2 ng·L^−1^) when LIN, SMZ, SMX, and STZ were detected. Notably, SDZ was detected in March, but not in June, and LIN was detected at all points except I-5, corroborating its intensive flow into the groundwater in June compared to the other months examined.

The concentrations of residual antibiotics in the groundwaters of I-2 increased 5.5 times in August compared to those recorded in June (35.0 and 5.5 ng·L^−1^, respectively; Figure 6c), primarily as a result of increasing SDZ and SMX concentrations (18.0 and 14.9 ng L^−1^, respectively). The observed increase in August in antibiotic concentrations in the groundwater of watershed I (including I-2) was likely driven by increasing precipitation, enhancing the percolation of antibiotics into the groundwater.

The cumulative averages of residual antibiotic concentrations during the three sampling campaigns are shown in Figure 6d, wherein average concentrations peaked at I-8 (59.3 ng·L^−1^), followed by I-2 (27.5 ng·L^−1^). The concentrations of residual antibiotics at I-8, I-15, and I-16 (corresponding to the downstream region of subwatershed I) were higher than those obtained at other points, as ≥1 targeted species were consistently detected. Paddy fields are distributed near I-8, I-15, and I-16 (Figure 1), which are subjected to treatments with compost and liquid manure obtained via livestock excreta. It might be possible that the antibiotics present within the compost and liquid manure were discharged to the environment through this process, ultimately flowing into the groundwater; however, groundwater was sampled from wells connected to houses, and antibiotics above a certain concentration were consistently detected at I-8 and I-15, suggesting the existence of a constant, steady-rate pollution source. Other sampling points apart from I-8 and I-15 were also located near farmlands, but revealed different trends, further supporting that the residual antibiotics found at these two sites were influenced by other sources in addition to agricultural activities. Groundwater concentrations were lower than those present in the surface water samples, and the types of antibiotics detected also differed. Within the stream measurements, LIN showed an average concentration of 52.7 ng·L^−1^ from March to August; however, groundwater concentrations were <10 ng·L^−1^ across all points during the same period, confirming that both the types and quantities of antibiotics in the surface water samples and groundwater samples varied with time, likely due to external factors such as seasonal climatic variations and patterns of use. The residual concentrations of antibiotics in groundwater were less sensitive to the effects of pollution sources than those of antibiotics in surface water. Therefore, it was estimated that pollution sources that emit antibiotics existed close to the sub-watershed.

### 3.4. Risk Assessment

#### 3.4.1. Ecological Risk Assessment of Surface Water Antibiotics

**Figure 7:**
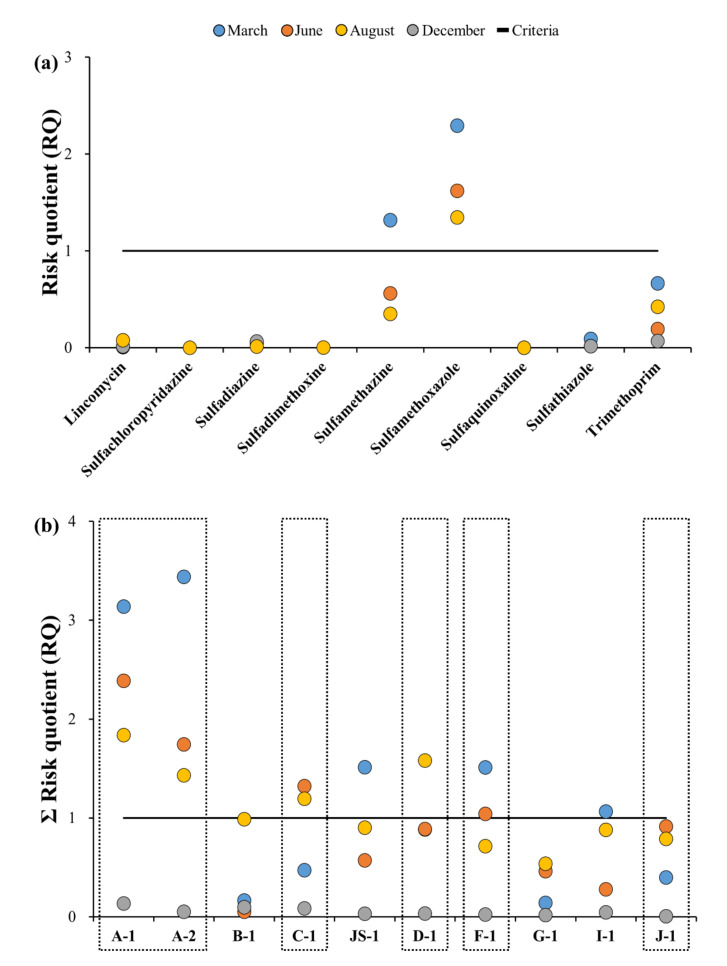
Ecological risk assessment of target antibiotics: (**a**) Risk quotient (RQ) for each sampling campaign and antibiotic. (**b**) RQ of each sampling campaign at each sampling point for surface water; Dotted line boxes: tributaries, Blue circle: March, Orange circle: June, Yellow: August, Grey: December, Solid line: Criteria (RQ = 1).

**Figure 8:**
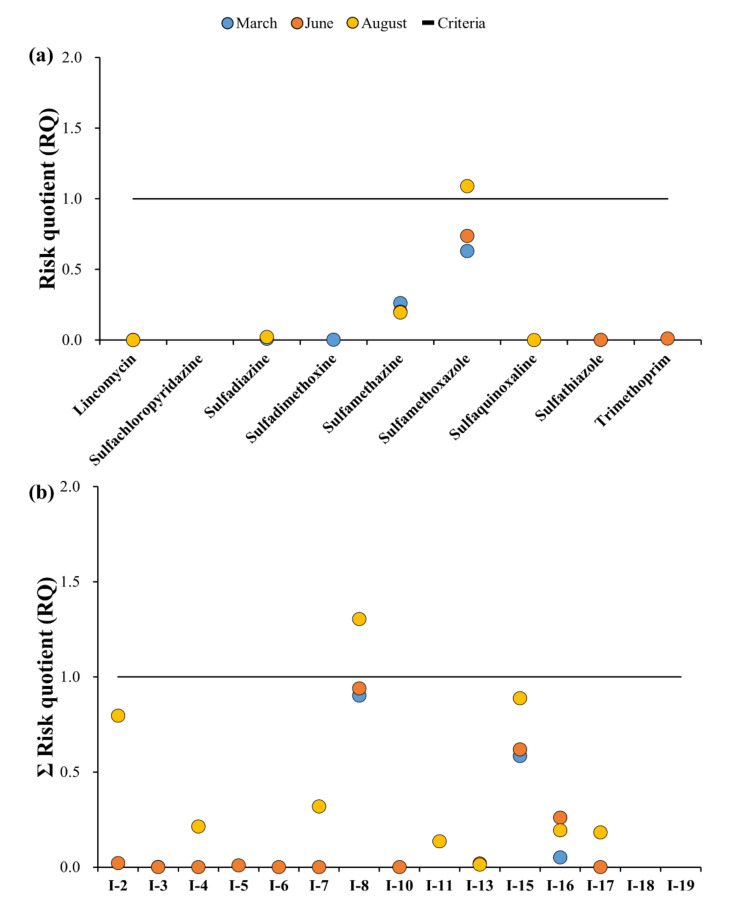
Ecological risk assessment of target antibiotics: (**a**) Risk quotient for each sampling campaign for each antibiotic. (**b**) RQ of each sampling campaign at each sampling point for groundwater; Blue circle: March, Orange circle: June, Yellow: August, Grey: December, Solid line: Criteria (RQ = 1).

The RQs were calculated to examine the ecological risks in the target stream (results are shown in Figure 7 and Figure 8, and further details are presented in Table A5). Figure 7a,b show the RQ values of antibiotics and sampling points for each sampling campaign. The PNEC values of the target antibiotics were obtained from the literature, and showed variations from 135 ng·L^−1^ for SDZ, to 131,000 ng·L^−1^ for SQX (Table A5) [3,39,43]. The highest RQ values of the antibiotics were selected to perform a conservative risk assessment (Figure 7a). The range and average RQ values of the nine antibiotics detected in the Cheongmi Stream watershed were 1.3 × 10^−5^–2.3, and 0.356, respectively, and these could be further classified as low (RQ < 0.1), medium (0.1 < RQ < 1), and high risk (RQ > 1) [44,45,46]. The RQ values of six of the ten antibiotics were considered low risk (<0.1); however, the RQs for SMZ, SMX, and TMP were relatively high, peaking in the spring and reaching its minimum value in the summer, likely resulting from the seasonal variations in the environmental concentrations of the antibiotics. SMZ demonstrated an RQ > 1 in March (1.3) only, whereas SMX presented with an RQ > 1 in March, June, and December (2.3, 1.6, and 1.4, respectively).

Since various antibiotics exist simultaneously within the environment, there is disconnection during evaluation of the risks of a location using the index of a single antibiotic; thus, ecological risks of the antibiotics in each location were evaluated by applying the concept of an additional effect (Figure 7b). If the sum of the RQs (ΣRQ) of each point obtained by applying the additional effect was <1, it was classified as no risk; in case it was between 1–10, it was classified as potentially hazardous; and in case it was >10, it was classified as the most hazardous. ΣRQ of the 10 sampling points ranged from 4.9 × 10^−3^ (J-1) to 3.4 (A-2), with no point classified as most hazardous; however, all points except for B-1, G-1, and J-1 were classified as potentially hazardous. At points A-1 and A-2, the ΣRQ was >1 for each month analyzed except August. In March, each of these points showed the highest ΣRQ values observed (3.1 and 3.4, respectively). ΣRQ was >1 at C-1 in June and December, whereas ΣRQ was >1 at F-1 in March and June. Thus, the periods where antibiotics showed the highest risk varied by location. The risks calculated in the present study depended on the region and time periods and tended to be high in regions where the environmental concentrations were high. Hence, it is believed that the environmental concentration plays an important role in the risk.

#### 3.4.2. Ecological Risk Assessment of Groundwater Antibiotics

The RQs indicating the ecological risk of the target antibiotics in the groundwater by sampling site and month are shown in Figure 8a,b, and detailed results can be found in Table A6. The same target antibiotic PNEC values were used when evaluating the ecological risks associated with the surface water.

**Figure A2:**
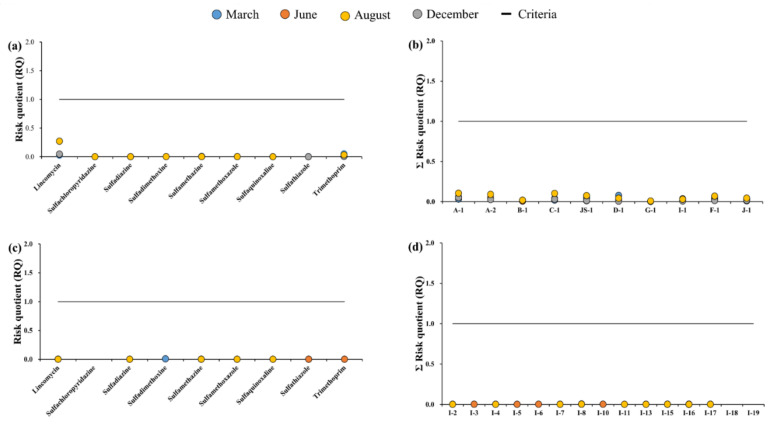
Risk assessment of antibiotic resistance by sampling time for surface water samples: (**a**) target antibiotics, and (**b**) sampling points; assessment for groundwater samples: (**c**) target antibiotics, and (**d**) sampling points.

Figure A2a shows the results of the maximum RQ values among the groundwater sampling points. The lowest observed RQ in the groundwater was recorded at SQX in August (7.6 × 10^−5^), whereas the maximum RQ was recorded at SMX in August (1.1). Unlike the surface water sample values, the groundwater samples of SMZ, SMX, and TMP showed RQ values > 1, with only SMX classified as high risk, and SMZ classified as medium risk. Thus, the antibiotics detected in the groundwater samples showed lower risks compared to those in the surface water samples. Furthermore, the maximum groundwater RQ observed was in August, whereas the surface water peak occurred in March.

The ΣRQ of the groundwater samples at each sampling point ranged from 6.6 × 10^−4^ (I-4, June) to 1.3 (I-8, August). Similar to that of the surface water samples, no sampling points were classified as the most hazardous (10 < ΣRQ). In addition to the August ΣRQ value at I-8, values recorded in March and June were also almost potentially hazardous (9.0 × 10^−1^ and 9.4 × 10^−1^, respectively); thus these levels did pose a considerable risk. The ΣRQ at I-15 was the second highest, but remained < 1 across all sampling campaigns; accordingly, it was found that all sampling points (except for I-8) were classified as points with no risk.

#### 3.4.3. Antibiotic Resistance-Based Risk Assessment in the Cheongmi Watershed

The antibiotic resistance-associated risks of the surface water samples and groundwater samples are shown in Figure A2a–d, and detailed risk values are listed in Table A7 and Table A8. The PNEC values of the target antibiotics were obtained from the literature and ranged from 500 to 1,000,000 ng·L^−1^, and values were notably higher than those applied in the ecological risk assessment [19,22,30].

The antibiotic resistance-based risk assessment for each target antibiotic in the surface water showed that SQX demonstrated the lowest RQ (6.5 × 10^−6^), whereas LIN showed the highest value (0.27; Figure A2a, Table A7). The RQs obtained for all other antibiotics were <0.1; thus, a vast majority of targeted antibiotics were classified as antibiotics with low risk. The RQs for each antibiotic detected in the groundwater were lower than those observed in surface water samples, ranging from 6.4 × 10^−6^ (SDZ) to 7.2 × 10^−3^ (SDX; Figure A2c). Accordingly, it was similarly determined that all antibiotics present in the groundwater posed low levels of risk.

Furthermore, the ΣRQ value at each surface water sampling point ranged from 2.9 × 10^−3^ to 0.11, reaching its maximum at A-1, and demonstrated minimum values at G-1 (Figure A2c). As ΣRQ was not >1 for all sampling points, the surface water in the Cheongmi Stream watershed could be considered to be not under any significant risk. The same trends were recorded for the groundwater samples, wherein the range of ΣRQ recorded in the Cheongmi Stream watershed was from 5.0 × 10^−4^ to 4.5 × 10^−3^ (Table A8). This suggested that although residual antibiotics were present in the Cheongmi Stream watershed, their levels were not significantly high enough to cause any significant antibiotic resistance-associated risk.

A comparison of the results of the present study with those of the antibiotic resistance-based risk assessment reported by Zhang et al. (2020) revealed that only LIN and TMP were classified as classes posing a medium risk in both studies, whereas the sulfonamides, SCP, SDZ, SMZ, SMX, STZ, and SQX were classified as classes posing low risks. In contrast, the calculated RQs reported herein were <1 for all sampling points in the Cheongmi Stream watershed; however, Zhang et al. (2020) found that the RQs of LIN and TMP were >1 in certain areas. The risk assessment revealed that the RQ, which was >1 and had a relatively larger value for PNEC for antibiotic resistance-based risk assessment than PNEC for ecological risk assessment. However, experimental determination of PNEC in complex microbial systems has limitations [22]. The PNEC for ecological risk assessment has been studied for a long time. In contrast, the data on PNEC for antibiotic resistance based risk assessment are scarce, so its reliability is relatively low. Therefore, more studies are required to identify the PNEC for antibiotic resistance-based risk assessment based on scientific data.

## 4. Conclusions

The present study was conducted to measure the residual antibiotic concentrations present in the surface (i.e., stream) and groundwater samples of an urban–rural complex watershed with intensive livestock farms in South Korea via simultaneous analyses using UHPLC-HRMS. The concentration and the detection frequency of antibiotics in the surface water were higher than those reported for the groundwater; however, differences in seasonal trends and concentrations were observed in both types of samples. In the surface water samples, concentrations were generally high in December, whereas concentrations of groundwater tended to peak in August. Therefore, the residual antibiotics in the surface waters of the watershed seemed to correlate with the pollution sources (e.g., effluent from individual treatment facilities for livestock excreta), climatic factors (e.g., precipitation), and human activities (e.g., use of antibiotics and agricultural activities). Thus, it was concluded that various factors can exert a direct impact on the residual antibiotic types and levels observed. Furthermore, relatively high concentrations of residual antibiotics were detected consistently in the groundwater, implying greater contamination from antibiotics use than agricultural activities, and such activities have been reported to be highly seasonal. This study confirmed that the concentration of antibiotics in groundwater was remarkably affected by spatial variations rather than by temporal effects. This study also performed ecological and antibiotic resistance risk assessments to examine the safety of the antibiotics detected in the surface- and groundwaters of the Cheongmi Stream watershed. Among the target antibiotics, many showed some level of risk based on the ecological risk assessment (RQ > 1); however, no risks pertaining directly to the targeted antibiotics were revealed. As antibiotics are an indispensable feature of modern human life and livestock breeding, their environmental release poses a significant risk to both ecological and human health, and they require constant biological monitoring through ecological risk assessments and antibiotic resistance risk assessments. Although antibiotics must be managed carefully to avoid promoting resistant strains of bacteria, the availability of relevant data on these aspects remains insufficient. Accordingly, the results obtained in the present study can lead to the improvement and establishment of future antibiotic risk assessments. Further studies on antibiotic resistance risk assessments should be conducted continuously while incorporating appropriate PNEC values. Moreover, the present study only confirmed the environmental residual concentration and risk; further research is required to comprehensively assess the environmental fate of antibiotics including their persistence, bioaccumulation, and mobility in the environment media.

## Figures and Tables

**Table 1:** Predicted no effective concentrations (PNECs) of the studied antibiotics.

	Ecological Risk Assessment ^a^	Antibiotic Resistance Risk Assessment
Lincomycin (LIN)	7000	2000 ^b^
Sulfachloropyridazine (SCP)	26,400	1,000,000 ^c^
Sulfadiazine (SDZ)	1350	2,000,000 ^c^
Sulfadimethoxine (SDX)	5290	1561 ^d^
Sulfamethazine (SMZ)	87	16,000 ^c^
Sulfamethoxazole (SMX)	19	16,000 ^b^
Sulfaquinoxaline (SQX)	131,000	256,000 ^c^
Sulfathiazole (STZ)	5000	256,000 ^c^
Trimethoprim (TMP)	40	500 ^b^

The values of PNEC for target antibiotics derived by ^a^ [5]; ^b^ [22]; ^c^ [19]; ^d^ [30].

**Figure A1:**
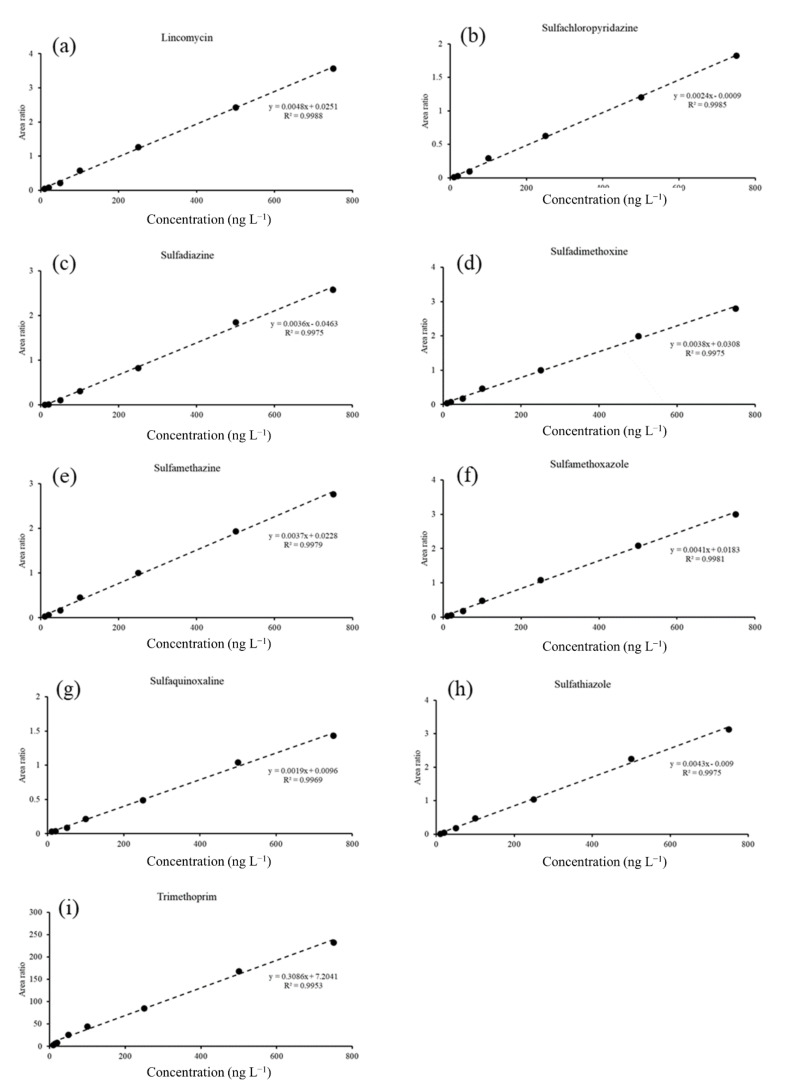
Calibration curves using UHPLC-q-orbitrap HRMS, combined with an online SPE analysis for the nine antibiotics analyzed. (**a**) lincomycin, (**b**) sulfachloropyridazine, (**c**) sulfadiazine, (**d**) sulfadimethoxine, (**e**) sulfamethazine, (**f**) sulfamethoxazole, (**g**) sulfaquinoxaline, (**h**) sulfathiazole, and (**i**) trimethoprim.

## Data Availability

Data available in a publicly accessible repository.

## Appendix A

Text A1. Chemicals and materials

The nine standard materials (lincomycin, LIN; sulfachloropyridazine, SCP; sulfadiazine, SDZ; sulfadimethoxine, SDX; SMZ; sulfamethoxazole, SMX; sulfaquinoxaline, SQX; sulfathiazole, STZ; and trimethoprim, TMP), and five ISTD materials (SDZ-13C6, SMZ-13C6, SMX-d4, SDX-d6, and TMP-d9) were purchased from Sigma-Aldrich (St. Louis, MO, USA), except for LIN-d3 and STZ-13C6, which were purchased from LGC Standards (Teddington, UK).

LC/MS grade methanol and acetonitrile used in the UHPLC-HRMS analysis were purchased from Fisher Scientific (Fairlawn, NJ, USA) and Merck Millipore (Billerica, MA, USA), respectively. Citric acid, trisodium citrate dehydrate, and ethylenediaminetetraacetic acid disodium salt dehydrate (EDTA) used in the sample pretreatment were purchased from Sigma-Aldrich (St. Louis, MO, USA), and formic acid was acquired from Honeywell Fluka (Morris Plains, NJ, USA).

Text A2. Surface water and groundwater sampling and sample preparation for analysis of antibiotic content.

Polypropylene bottles (2 and 1 L) were used after rinsing with surface water or groundwater 3–4 times. Surface water was sampled at ~0.1 m below the water surface. Water samples were stored under dark conditions at <4 °C until analysis. To remove suspended solids, the samples were centrifuged at 4 °C and 24,600× *g* for 5 min. Subsequently, the supernatant was filtered using a 0.2-μm polyvinylidene fluoride syringe filter, and the pH of the sample was adjusted to 6.5 using 0.5 mL of 1.5 M citrate buffer solution per 50 mL of sample, following which 5% EDTA solution (0.5 mL per 50 mL of sample) was added. Finally, 25 mL (500 ng·L^−1^) of a mixture solution containing seven ISTDs was added to the 50-mL sample. The pre-treated samples were analyzed within seven days.

Text A3. Analysis of the surface water and groundwater samples.

Hypersil GOLD aQ and Reversed Xbridge C18 columns (2.1 mm × 50 mm i.d., 2.5 μm, Waters, Milford, MA, USA) were used to extract and analyze the antibiotics present in the samples, respectively. Total analysis time was 30 min, and 5 mL of the sample was injected over a duration of 5 min. The detailed equipment conditions for antibiotics are listed in Table A1. The Xcalibur V. 4.0 (Thermo Fisher Scientific, Bremen, Germany) software was used for analysis of the control sample and for data collection.

Text A4. Quality assurance and control considered for surface water and groundwater sample data.

Each sample was analyzed three times, and the blanks and solvent blanks were analyzed after every analysis of six samples. The calibration curve was obtained using the results obtained for seven solutions in the range of 10–750 ng·L^−1^, with each solution containing 250 ng·L^−1^ of the ISTD mixture solvent. The coefficient of determination (R^2^) for each antibiotic was estimated to be >0.99 (Figure A1). The method detection limit (MDL) and method quantification limit (MQL) of the antibiotics were determined by multiplying the standard deviation value obtained by analyzing the 10 ng·L^−1^ standard solution seven times, by 3.14 and 10, respectively (Table A2).

**Table A1:** Instrument conditions for the selected veterinary antibiotics.

SPE Column		Hypersil GOLD aQ (2.1 × 20 mm; particle size, 12 μm) (Thermo Scientific Fisher, Bremen, Germany)					
HPLC Column		XBridge C18 column (2.1 mm × 50 mm i.d., 2.5 μm, Waters, Milford, MA, USA)					
Injection Volume (μL)		5000					
Mobile Phase		(A) 0.1% formic acid in DW					
		(B) Acetonitrile					
Chromatography Condition							
UHPLC				Online SPE			
Time (min)	Flow rate (mL·min^−1^)	A (%)	B (%)	Time (min)	Flow rate (mL·min^−1^)	A (%)	B (%)
0	0.2	98	2	0	1	98	2
5.1		98	2	5.1	1		
10		35	65	5.2	0.1		
18.5		35	65	27.3	0.1		
26		2	98	27.4	1		
26.1		98	2	30	1		
30		98	2				
Orbitrap MS Condition							
Ionization mode	Positive	Resolving power		17,500 FWHM (*m*/*z* 200)	Flow rate of sheath gas	45 arb	
Spray voltage	3.5 kV	Automatic gain control (AGC)		5 × 10^5^	Flow rate of sweep gas	2 arb	
Capillary temperature	250	Maximum injection time (IT)		100 ms	Flow rate of auxiliary gas	10 arb	
Auxiliary gas temperature	400	Normalized collision energy (NCE)		30			

**Table A2:** Method detection limit (MDL) and method quantification limit (MQL) of the nine target antibiotics.

Chemical Name	Method Detection Limit (ng·L^−1^)	Method Quantification Limit (ng·L^−1^)
Lincomycin (LIN)	0.44	1.40
Sulfachloropyridazine (SCP)	0.36	1.16
Sulfadiazine (SDZ)	1.59	5.07
Sulfadimethoxine (SDX)	0.48	1.53
Sulfamethazine (SMZ)	0.52	1.66
Sulfamethoxazole (SMX)	0.89	2.82
Sulfaquinoxaline (SQX)	0.67	2.15
Sulfathiazole (STZ)	1.07	3.41
Trimethoprim (TMP)	0.44	1.41

**Table A3:** Residual concentrations of antibiotics in the surface water samples (ng·L^−1^). Blank cells indicate samples where no antibiotic could be detected.

		LIN	SCP	SDZ	SDX	SMZ	SMX	SQX	STZ	TMP
March	A-1	31.1		29.4		114.6	31.6		218.3	3.5
	A-2	35.8		44.2		83.7	43.6		96.1	5.1
	B-1	0.8		15.8				12.1	457.0	2.5
	C-1	37.8		35.0		7.5	6.7			
	D-1	48.8		60.7		3.5	2.4			26.6
	JS-1	39.0		83.3		30.5	14.8		8.1	12.6
	F-1	34.6		29.1		42.2	13.0		18.1	12.5
	G-1	6.4		16.1		11.1				
	I-1	22.5		33.6		28.7	8.2			11.0
	J-1	8.0		33.3			5.8			2.6
June	A-1	79.3				48.9	30.8		6.0	7.7
	A-2	68.4		33.2		44.0	20.3			5.3
	B-1	13.4				4.1		1.7	8.5	
	C-1	54.1				34.2	15.5			4.3
	D-1	32.0		46.8		16.7	11.4			2.3
	JS-1	11.5		32.8		3.3	8.6			2.2
	F-1	35.8		34.2		18.6	13.5		81.2	2.8
	G-1	6.5		39.9		5.8	6.9			
	I-1	14.5		59.0		5.4				6.8
	J-1	28.8		32.6		12.7	12.4			3.4
August	A-1	94.4		87.8					82.9	1.6
	A-2	53.4		52.9					19.3	
	B-1	24.0		28.2					14.7	2.8
	C-1	55.9		38.0					25.0	1.8
	D-1	11.7		43.4						
	JS-1	26.5		32.2					17.0	
	F-1	26.9		27.1						
	G-1	5.8		23.3						
	I-1	15.6		58.4						
	J-1	33.9								
December	A-1	463.9		16.4	5.8	23.1	20.3	5.0		16.9
	A-2	438.0			3.3	30.4	19.4			
	B-1	6.0	8.8		4.7	10.2	12.4	3.9		8.7
	C-1	542.0			4.8	26.0	10.0			11.6
	D-1	30.1	4.6	17.6	4.7	5.5	25.6	26.4		6.1
	JS-1	363.5	6.5		4.9	17.9	9.1	7.6		6.7
	F-1	291.2			5.6	22.2	5.3	5.7		5.5
	G-1	8.2				30.2	2.7			1.8
	I-1	13.6	5.3	17.7	8.6	25.9	5.9			10.2
	J-1	190.7			4.7	21.8	6.0	5.7		7.9
Average 2018	A-1	167.1		48.1	5.8	62.2	27.6	5.0	129.9	7.1
	A-2	148.9		43.4	3.3	52.7	28.7		57.7	5.2
	B-1	11.0	8.8	22.0	4.7	7.1	12.4	6.8	202.5	4.9
	C-1	172.5		36.5	4.8	22.6	10.6		25.0	6.4
	D-1	30.6	4.6	42.1	4.7	8.6	14.9	26.4		12.4
	JS-1	110.1	6.5	49.4	4.9	17.2	10.8	7.6	11.6	8.6
	F-1	103.5		30.5	5.6	27.7	10.2	5.7	49.6	6.9
	G-1	6.7		24.7		15.7	4.1			1.8
	I-1	16.6	5.3	44.4	8.6	20.0	7.0			9.2
	J-1	65.4		32.9	4.7	17.2	7.8	5.7		4.6

**Table A4:** Residual concentrations of antibiotics in the groundwater samples (ng L^ࢤ1^). Blank cells indicate samples where no antibiotic could be detected.

		LIN	SCP	SDZ	SDX	SMZ	SMX	SQX	STZ	TMP
March	I-2									
	I-3				11.2					
	I-4									
	I-5									
	I-6									
	I-7									
	I-8			12.8		22.7	12.0		8.8	
	I-10									
	I-11									
	I-13									
	I-15					1.6	10.8			
	I-16				2.3		1.0			
	I-17									
	I-18									
	I-19									
June	I-2	4.6				1.8				
	I-3	4.6								
	I-4	4.7								
	I-5									0.4
	I-6	4.7								
	I-7	4.8								
	I-8	5.1				17.5	14.0		6.7	
	I-10	4.6								
	I-11									
	I-13	4.7				1.6				
	I-15	4.7				1.7	11.4			
	I-16	4.7					4.9			
	I-17	4.6								
	I-18									
	I-19									
August	I-2	0.9		18.0			14.9	1.2		
	I-3									
	I-4	1.0					4.1	0.6		
	I-5									
	I-6									
	I-7	1.1					6.1	0.8		
	I-8	1.6		28.4		16.9	20.7	1.3		
	I-10									
	I-11	0.8					2.6	2.4		
	I-13	1.1		17.6				4.6		
	I-15	1.0				2.1	16.4	5.5		
	I-16	1.1					3.7	9.9		
	I-17	0.8					3.5	2.8		
	I-18									
	I-19									
Average 2018	I-2	5.5		18.0		1.8	14.9	1.2		
	I-3	4.6			11.2					
	I-4	5.7					4.1	0.6		
	I-5									0.4
	I-6	4.7								
	I-7	5.8					6.1	0.8		
	I-8	6.7		41.2		57.1	46.7	1.3	15.5	
	I-10	4.6								
	I-11	0.8					2.6	2.4		
	I-13	5.7		17.6		1.6		4.6		
	I-15	5.7				5.3	38.6	5.5		
	I-16	5.8			2.3		9.6	9.9		
	I-17	5.5					3.5	2.8		
	I-18									
	I-19									

**Table A5:** Ecological risk assessment for antibiotics in the surface water samples during each sampling campaign. Blank cells indicate samples where no antibiotic could be detected.

	**LIN**	**SCP**	**SDZ**	**SDX**	**SMZ**	**SMX**	**SQX**	**STZ**	**TMP**	
March	7.0 × 10^−3^		6.2 × 10^−2^		1.3	2.3	9.2 × 10^−5^	9.1 × 10^−2^	6.7 × 10^−1^	
June	1.1 × 10^−2^		4.4 × 10^−2^		5.6 × 10^−1^	1.6	1.3 × 10^−5^	1.6 × 10^−2^	1.9 × 10^−1^	
August	1.3 × 10^−2^		6.5 × 10^−2^					1.7 × 10^−2^	6.9 × 10^−2^	
December	7.7 × 10^−2^	3.3 × 10^−4^	1.3 × 10^−2^	1.6 × 10^−^^3^	3.5 × 10^−1^	1.3	2.0 × 10^−4^		4.2 × 10^−1^	
	**A-1**	**A-2**	**B-1**	**C-1**	**JS-1**	**D-1**	**F-1**	**G-1**	**I-1**	**J-1**
March	3.1	3.4	1.6 × 10^−1^	4.7 × 10^−1^	1.5	8.8 × 10^−1^	1.5	1.4 × 10^−1^	1.1	4.0 × 10^−1^
June	2.4	1.7	5.0 × 10^−2^	1.3	5.7 × 10^−1^	8.9 × 10^−1^	1.0	4.6 × 10^−1^	2.8 × 10^−1^	9.1 × 10^−1^
August	1.3 × 10^−1^	5.1 × 10^−2^	9.6 × 10^−2^	8.5 × 10^−2^	3.1 × 10^−2^	3.4 × 10^−2^	2.4 × 10^−2^	1.8 × 10^−2^	4.5 × 10^−2^	4.8 × 10^−3^
December	1.8	1.4	9.9 × 10^−1^	1.2	9.0 × 10^−1^	1.6	7.1 × 10^−1^	5.4 × 10^−1^	8.8 × 10^−1^	7.9 × 10^−1^

**Table A6:** Ecological risk assessment for antibiotics in the groundwater samples during each sampling campaign. Blank cells indicate samples where no antibiotic could be detected.

	March	June	August		March	June	August
LIN		7.2 × 10^−^^4^	2.3 × 10^−^^4^	I-2		2.1 × 10^−^^2^	8.0 × 10^−^^1^
SCP				I-3	2.1 × 10^−^^3^	6.6 × 10^−^^4^	
SDZ	9.5 × 10^−^^3^		2.1 × 10^−^^2^	I-4		6.7 × 10^−^^4^	2.1 × 10^−^^1^
SDX	2.1 × 10^−^^3^			I-5		1.0 × 10^−^^2^	
SMZ	2.6 × 10^−^^1^	2.0 × 10^−^^1^	1.9 × 10^−^^1^	I-6		6.6 × 10^−^^4^	
SMX	6.3 × 10^−^^1^	7.4 × 10^−^^1^	1.1	I-7		6.8 × 10^−^^4^	3.2 × 10^−^^1^
SQX			7.5 × 10^−^^5^	I-8	9.0 × 10^−^^1^	9.4 × 10^−^^1^	1.3
STZ	1.8 × 10^−^^3^	1.3 × 10^−^^3^		I-10		6.6 × 10^−^^4^	
TMP		1.0 × 10^−^^2^		I-11			1.4 × 10^−^^1^
				I-13		1.9 × 10^−^^2^	1.3 × 10^−^^2^
				I-15	5.8 × 10^−^^1^	6.2 × 10^−^^1^	8.9 × 10^−^^1^
				I-16	5.1 × 10^−^^2^	2.6 × 10^−^^1^	1.9 × 10^−^^1^
				I-17		6.6 × 10^−^^4^	1.8 × 10^−^^1^
				I-18			
				I-19			

**Table A7:** Risk assessment for resistance to antibiotics in the surface water samples. Blank cells indicate samples where no antibiotic could be detected.

	**LIN**	**SCP**	**SDZ**	**SDX**	**SMZ**	**SMX**	**SQX**	**STZ**	**TMP**	
March	2.4 × 10^−2^		4.2 × 10^−5^		7.2 × 10^−3^	2.7 × 10^−3^	4.7 × 10^−5^	1.8 × 10^−3^	5.3 × 10^−2^	
June	4.0 × 10^−2^		2.9 × 10^−5^		3.1 × 10^−3^	1.9 × 10^−3^	6.5 × 10^−6^	3.2 × 10^−4^	1.5 × 10^−2^	
August	4.7 × 10^−2^		4.4 × 10^−5^					3.2 × 10^−4^	5.5 × 10^−3^	
December	2.7 × 10^−1^	8.8 × 10^−6^	8.9 × 10^−6^	1.6 × 10^−3^	1.9 × 10^−3^	1.6 × 10^−3^	1.0 × 10^−4^		3.4 × 10^−2^	
	**A-1**	**A-2**	**B-1**	**C-1**	**JS-1**	**D-1**	**F-1**	**G-1**	**I-1**	**J-1**
March	3.3 × 10^−^^2^	3.6 × 10^−^^2^	7.1 × 10^−3^	2.0 × 10^−^^2^	4.8 × 10^−^^2^	7.8 × 10^−^^2^	3.9 × 10^−^^3^	3.6 × 10^−^^2^	4.6 × 10^−^^2^	9.5 × 10^−^^3^
June	6.0 × 10^−^^2^	4.9 × 10^−^^2^	7.0 × 10^−3^	3.9 × 10^−^^2^	1.1 × 10^−^^2^	2.2 × 10^−^^2^	4.1 × 10^−^^3^	2.1 × 10^−^^2^	2.6 × 10^−^^2^	2.3 × 10^−^^2^
August	5.1 × 10^−^^2^	2.7 × 10^−^^2^	1.8 × 10^−^^2^	3.2 × 10^−^^2^	1.3 × 10^−^^2^	5.9 × 10^−^^3^	2.9 × 10^−^^3^	7.8 × 10^−^^3^	1.3 × 10^−^^2^	1.7 × 10^−^^2^
December	1.0 × 10^−^^1^	9.1 × 10^−^^2^	1.8 × 10^−^^2^	1.0× 10^−^^1^	7.5 × 10^−^^2^	4.3 × 10^−^^2^	8.2 × 10^−^^3^	3.0 × 10^−^^2^	6.9 × 10^−^^2^	4.4 × 10^−^^2^

**Table A8:** Risk assessment for resistance to antibiotics in the groundwater samples. Blank cells indicate samples where no antibiotic could be detected.

	March	June	August		March	June	August
LIN		2.5 × 10^−^^3^	8.1 × 10^−^^4^	I-2		2.4 × 10^−^^3^	1.4 × 10^−^^3^
SCP				I-3	2.1 × 10^−^^3^	2.3 × 10^−^^3^	
SDZ	6.4 × 10^−^^6^		1.4 × 10^−^^5^	I-4		2.3 × 10^−^^3^	7.7 × 10^−^^4^
SDX	7.2 × 10^−^^3^			I-5		8.1 × 10^−^^4^	
SMZ	1.4 × 10^−^^3^	1.1 × 10^−^^3^	1.1 × 10^−^^3^	I-6		2.3 × 10^−^^3^	
SMX	7.5 × 10^−^^4^	8.8 × 10^−^^4^	1.3 × 10^−^^3^	I-7		2.4 × 10^−^^3^	9.2 × 10^−^^4^
SQX			3.9 × 10^−^^5^	I-8	2.2 × 10^−^^2^	4.5 × 10^−^^3^	3.2 × 10^−^^3^
STZ	3.4 × 10^−^^5^	2.6 × 10^−^^5^		I-10		2.3 × 10^−3^	
TMP		8.1 × 10^−^^4^		I-11			5.9 × 10^−^^4^
				I-13		2.4 × 10^−^^3^	5.6 × 10^−^^4^
				I-15	7.7 × 10^−^^4^	3.2 × 10^−^^3^	1.7 × 10^−^^3^
				I-16	5.0 × 10^−^^4^	2.7 × 10^−^^3^	8.3 × 10^−^^4^
				I-17		2.3 × 10^−^^3^	6.3 × 10^−^^4^
				I-18			
				I-19			
